# Identification of Two Linear Epitopes on MGF_110-13L Protein of African Swine Fever Virus with Monoclonal Antibodies

**DOI:** 10.3390/ani14131951

**Published:** 2024-07-01

**Authors:** Shu-Jian Zhang, Bei Niu, Shi-Meng Liu, Yuan-Mao Zhu, Dong-Ming Zhao, Zhi-Gao Bu, Rong-Hong Hua

**Affiliations:** 1 State Key Laboratory of Animal Disease Control and Prevention, Harbin Veterinary Research Institute of Chinese Academy of Agricultural Sciences, Harbin 150069, China; 2 Jiangsu Co-Innovation Center for Prevention and Control of Important Animal Infectious Diseases and Zoonoses, Yangzhou University, Yangzhou 225009, China

**Keywords:** African swine fever virus, MGF_110-13L protein, monoclonal antibody, epitope

## Abstract

**Simple Summary:**

Due to the complexity of the structure of African swine fever virus (ASFV), the structure and function of most viral encoded proteins are uncharacterized. A full understanding and identification of ASFV-coding proteins is conducive to the prevention and control of ASFV. In this study, a previously uncharacterized protein, MGF_110-13L, was identified as an immunogenic protein and expressed as a glycosylated homodimer in eukaryotic cells. Two monoclonal antibodies against MGF_110-13L were generated, and two linear epitopes were further identified using monoclonal antibodies. One epitope peptide was evaluated as a potential antigen for ASFV antibody detection. This study will provide a basis for further understanding the structure and function of the MGF_110-13L protein of ASFV.

**Abstract:**

African swine fever caused by African swine fever virus (ASFV) is an acute, highly contagious swine disease with high mortality. To facilitate effective vaccine development and find more serodiagnostic targets, fully exploring the ASFV antigenic proteins is urgently needed. In this study, the MGF_110-13L was identified as an immunodominant antigen among the seven transmembrane proteins. The main outer-membrane domain of MGF_110-13L was expressed and purified. Two monoclonal antibodies (mAbs; 8C3, and 10E4) against MGF_110-13L were generated. The epitopes of two mAbs were preliminary mapped with the peptide fusion proteins after probing with mAbs by enzyme-linked immunosorbent assay (ELISA) and Western blot. And the two target epitopes were fine-mapped using further truncated peptide fusion protein strategy. Finally, the core sequences of mAbs 8C3 and 10E4 were identified as ^48^WDCQDGICKNKITESRFIDS^67^, and ^122^GDHQQLSIKQ^131^, respectively. The peptides of epitopes were synthesized and probed with ASFV antibody positive pig sera by a dot blot assay, and the results showed that epitope 10E4 was an antigenic epitope. The epitope 10E4 peptide was further evaluated as a potential antigen for detecting ASFV antibodies. To our knowledge, this is the first report of antigenic epitope information on the antigenic MGF_110-13L protein of ASFV.

## 1. Introduction

ASF is a highly contagious and severe hemorrhagic swine disease caused by ASFV. Both domestic and wild boars are susceptible to ASFV. The World Organization for Animal Health (WOAH) has listed ASF as a notifiable disease due to the severe pathogenicity of ASFV and its ability to spread rapidly. Since its first description in Kenya in 1921, ASF has subsequently spread throughout sub-Saharan Africa [1]. In addition, there have been several transcontinental spreads of the disease, such as when it was introduced into the Caucasus region and Eastern Europe in 2007 [2], and emerged in China and Southeast Asia in 2018 [3,4,5]. Worldwide, the ASF outbreak and epidemic have resulted in huge economic losses as well as negative social consequences. There is currently no reliable commercial vaccine for ASF. Currently, the only effective control and eradication measures are strict stamping-out policies based on early detection.

ASFV belongs to and is the only member of the family *Asfarviridae* and the genus *Asfivirus* [6]. The linear, double-stranded DNA genome is approximately 170–190 kbp in size, with over 150 open reading frames, including more than 50 structural proteins and over 100 nonstructural proteins [6,7,8]. However, the structural and functional properties of the majority of ASFV proteins are unknown, which has hampered research into ASF vaccines and diagnostics. Till now, it has been known that some structural proteins of ASFV, such as P72, P30, and P54, are highly immunogenic. These proteins have been extensively used as immunoserological diagnosis targets and subunit vaccine components [9,10,11,12,13]. In addition to these major structural proteins, there are proteins in the ASFV-encoded proteomic that have good antigenicity and potential value in inducing immune protection and serological diagnosis. For example, the nonstructural protein K205R is a serological immunodeterminant of ASFV [14,15,16]. The immunogenic K205R protein stimulates a robust antibody response in animals that have been immunized, no matter whether it is expressed prokaryotically or eukaryotically [17,18]. To facilitate ASFV subunit vaccine research and explore new serodiagnostic targets, it is necessary to fully explore the antigenic proteins in the ASFV proteome. In particular, because the ASFV possesses a multilayered structure [19,20], the inner and outer membrane proteins are not fully characterized.

In the present study, using ASFV antibody positive pig sera, we identified MGF_113-13L as a novel antigenic protein from seven uncharacterized transmembrane proteins. Using mammalian cell line expressing MGF_110-13L protein as an immunogen, we generated two MGF_110-13L protein-specific monoclonal antibodies (mAbs). The target epitopes of these mAbs were fine-mapped and the potential application of the epitope peptides as serodiagnostic antigens was evaluated.

## 2. Materials and Methods

### 2.1. Cells, Sera, and Viruses

SP2/0 cells (ATCC, CRL-1581) were maintained in RPMI (Gibco, Beijing, China); baby hamster kidney BHK-21 cells (ATCC, CCL-10) and human embryo kidney 293 HEK-293 cells (ATCC, CRL-1573) were maintained in Dulbecco’s modified Eagle medium (DMEM; Gibco, Beijing, China). All cell media were supplemented with 10% fetal bovine serum, 100 µg/mL streptomycin and 100 U/mL penicillin. All cells were maintained in a humidified 5% CO_2_ atmosphere at 37 °C. The ASFV (HLJ/18 strain) was propagated and titrated in primary porcine alveolar macrophages (PAMs) as described [4]. As stated in a prior study [21], pig sera against ASFV were obtained from pigs immunized with the attenuated ASFV strain HLJ/18-7GD, either with or without challenge with the virulent ASFV strain HLJ/18. The Diagnostic Center of Harbin Veterinary Research Institute provided the ASFV antibody-positive serum samples from pigs naturally ASFV-infected. Specific pathogen-free (SPF) pig serum was used as a negative control.

### 2.2. Plasmids Construction and Transient Transfection

According to the TMHMM transmembrane helix prediction (https://services.healthtech.dtu.dk/services/TMHMM-2.0/, accessed on 1 February 2020), the main outer-membrane domains of seven ASFV transmembrane proteins were selected for transient expression in HEK-293 cells. The main outer-membrane domains were 1 to 160 amino acids of C257L protein, 1 to 237 amino acids of I329L protein, 22 to 177 amino acids of I177L protein, 47 to 158 amino acids of EP153R protein, 29 to 475 amino acids of B475L protein, 35 to 196 amino acids of K196R protein, and 1 to 131 amino acids of MGF_110-13L protein, respectively. All protein sequences were referenced to ASFV strain HLJ/18 in the African Swine Fever Virus Database (http://asfvdb.popgenetics.net/index, accessed on 6 January 2020). The genetic codon-optimized cDNAs encoding the respective outer-membrane domains were synthesized and cloned into the expression vector pCAGneo with a 6× His tag at the carboxyl terminal [18,22]. DNA sequencing was used to verify all of the recombinant plasmids. The plasmids were transfected into HEK-293 cells with ExFect Transfection Reagent (Vazyme, Nanjing, China). After 48 h of transfection, the protein samples were collected from the transfected cells for Western blot analysis.

### 2.3. Expression and Purification of the MGF_110-13L Protein

Stable expression cell lines were constructed as previously described [18,22]. Briefly, the ectodomain of MGF_110-13L (amino acid positions 1 to 131, reference sequence: QBH90491) expression plasmids were linearized with SspI. Then, BHK-21 cells were transfected with linearized plasmids. Transfected cells were cloned and selected using G418. The engineered cell clones were tested using an indirect immunofluorescence assay (IFA) and Western blot with a His tag-specific mouse monoclonal antibody. One clone, designated BHK-110-13L, demonstrated more efficient expression of the recombinant protein and was thus chosen and maintained in G418-supplemented medium for further characterization and production. The His-tagged recombinant protein was purified from the supernatants of the stable expression cell line via affinity chromatography with an Ni-NTA agarose column. The protein concentration was determined using a BCA protein assay kit (Biosharp, Hefei, China). Purified proteins were stored at −80 °C until use.

### 2.4. Generation of Monoclonal Antibodies against MGF_110-13L

Monoclonal antibodies against MGF_110-13L protein were generated as previously described [16,23]. Briefly, BALB/c mice were immunized subcutaneously with purified protein emulsified with Freund’s adjuvant (Sigma, St. Louis, MO, USA) three times at an interval of 21 days. A final booster immunization by intraperitoneal injection with soluble 13L-His protein alone was given 3 days before the cell fusion. Hybridomas were obtained by fusing mouse splenocytes with SP2/0 cells. Hybridomas were screened by ELISA, and positive cell lines were subcloned by limiting dilution method. Monoclonal antibodies were purified using affinity chromatography with a Protein G column (Abbkine, Wuhan, China). Each mAb’s heavy and light chains were identified using a mouse antibody isotyping kit (Thermo Scientific, Rockford, IL, USA).

### 2.5. Immunofluorescence Assay

Indirect immunofluorescence assays were carried out as previously described [16]. Briefly, the MGF_110-13L expressing cells were fixed and permeabilized. After blocking with BSA, the cells were incubated with His tag specific antibodies. After washing, the cells were incubated with fluorescein-conjugated goat anti-mouse IgG. Nuclei were stained by 4′,6-diamidino-2-phenylindole (DAPI). Finally, after three PBS washes, the cells were visualized using fluorescence microscopy. Untransfected BHK-21 cells were used as the negative control.

### 2.6. Expression of Short Peptide Fusion Proteins

Short peptides were expressed as fusion proteins with maltose-binding protein (MBP) as previously described [16,24]. Briefly, peptide-encoding DNA fragments were synthesized and cloned into plasmid pMAL-c5X between the sites of NdeI and BamHI. After being verified by DNA sequencing, the recombinant plasmids were transformed into expressing host *E. coli* ER2523. After inducing with isopropyl-beta D-thiogalactopyranoside (IPTG), the cell lysates were analyzed with sodium dodecyl sulfate-polyacrylamide gel electrophoresis (SDS-PAGE).

### 2.7. SDS-PAGE and Western Blotting

Lysates of bacterial-expressed peptide fusion proteins or ASFV-infected cell lysates were resolved on 4–20% SDS-PAGE (GenScript, M42015C), then transferred onto nitrocellulose membranes and blocked with 5% skimmed milk at 4 °C for an overnight period. After that, the membranes were incubated for 1 h at 37 °C with either the specific monoclonal antibody or pig serum. After three washes with PBS plus 0.5% Tween-20 (PBST), the membranes were then incubated for 1 h at 37 °C with either horseradish peroxidase (HRP)-conjugated goat anti-pig IgG or anti-mouse Alexa Fluor 680-conjugated secondary antibodies (Thermo, A10038). The blots were developed with ECL substrate or visualized using the Li-Cor Odyssey system (Li-Cor Biosciences, Lincoln, NE, USA).

### 2.8. ELISA

ELISA tests were performed as previously described [16,24]. Briefly, microplates were coated with MBP-peptide fusion proteins and blocked with skimmed milk. After three washes, the plates were incubated with hybridoma supernatants. After washing, the plates were incubated with HRP-conjugated goat anti-mouse IgG (ZSGB-BIO, Beijing, China). Following three washes, the plates were developed with 3,3′,5,5′-tetramethyl benzidine (TMB) and stopped with 2 M H_2_SO_4_. The optical density was then measured with an ELISA plate reader.

### 2.9. Dot Blot Assay

The epitope peptides were chemically synthesized (GenScript, Nanjing, China) and dissolved in dH_2_O or DMSO according to the manufacturer’s instructions. Dot blot assays were performed as previously described [16]. Briefly, synthesized peptides were spotted onto the nitrocellulose membranes and incubated with pig sera. After washing, the membranes were incubated with HRP-conjugated goat anti-pig IgG. Then, after three washes, the blots were developed with ECL substrate and visualized with a chemilluminescence imaging system.

### 2.10. Immunization of Pigs

Six landrace pigs were randomly divided into two groups, with three pigs in each group. These pigs were approximately 30 days of age and had been determined to be ASFV antibody-negative prior to the immune experiment. Each pig in group one was immunized with purified MGF_110-13L protein at a dose of 100 μg protein emulsified with oil adjuvant. Twenty eight days after the first immunization, the pigs were given a booster immunization at the same dose. Blood samples were collected prior to and at 14, 28, and 42 days post-immunization. Another group of pigs served as a control group and were injected with the same volume of PBS each time. The antibody titers of pig sera were examined by ELISA as described in Section 2.8, with the exception that the coating antigen was purified MGF_110-13L protein and the secondary antibody was HRP-conjugated goat anti-swine IgG.

## 3. Results

### 3.1. MGF_110-13L Was Identified as an Antigenic Protein

**Figure 1:**
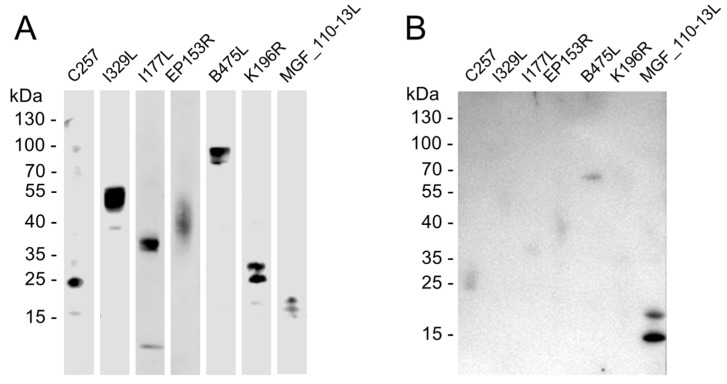
Identification of antigenic transmembrane proteins with pig anti-ASFV serum. Seven predicted ASFV transmembrane protein outer-membrane domain-expressing plasmids were transiently transfected into the HEK-293 cells. The protein expression was verified with 6× His tag specific antibody by Western blot (**A**). After verification, transiently expressed proteins were probed with pig anti-ASFV serum from pigs vaccinated with attenuated ASFV and challenged with a virulent ASFV strain (**B**).

To explore ASFV genome-encoded antigenic proteins, seven predicted transmembrane proteins were selected to express in mammalian cells. The main outer-membrane domain expression plasmids were constructed and expressed in mammalian cells by transient transfection. The expression of constructs was verified by Western blot analysis with 6× His tag-specific antibody. The results demonstrated that all seven constructs were expressed in transfected cells (Figure 1A). The transfected cell lysates were then probed with ASFV-infected pig serum by Western blot. As shown in Figure 1B, among the seven transmembrane proteins, only MGF_110-13L exhibited a strong blotting signal. These results suggested that MGF_110-13L was antigenic, which could induce antibody responses and be recognized by ASFV-infected pig serum.

### 3.2. Expression of MGF_110-13L Protein and Generation of Monoclonal Antibodies

**Figure 2:**
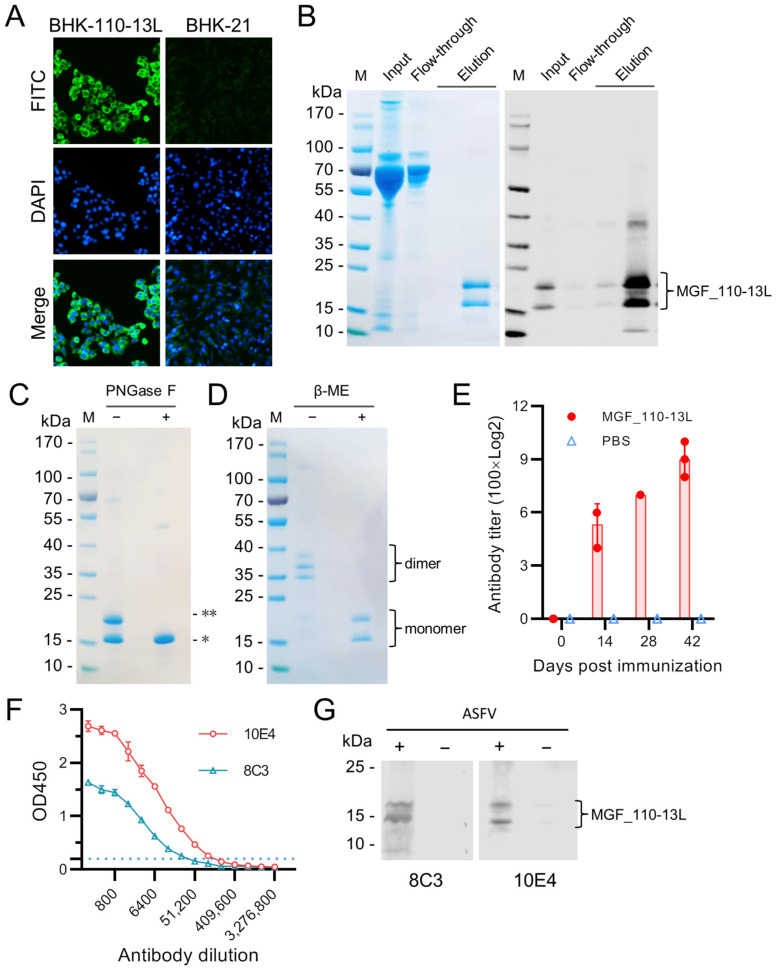
Expression of MGF_110-13L protein and generation of mAbs against the MGF_110-13L protein of ASFV. After transfection and selection, a stable cell line expressing the MGF_110-13L protein was established. The cell line was identified by IFA with 6× His tag specific antibody (**A**). The recombinant protein was purified by nickel affinity chromatography from the supernatants of the cell line and analyzed by SDS-PAGE and Western blot (**B**). The purified recombinant protein was treated with PNGase F and analyzed by SDS-PAGE (**C**). The purified proteins were analyzed by SDS-PAGE under non-reducing conditions (without β-mercaptoethanol) and reducing conditions (with β-mercaptoethanol) (**D**). The titers of MGF_110-13L-specific antibody in immunized pig sera were detected by ELISA (**E**). The titrations of monoclonal antibodies 8C3 and 10E4 were detected by ELISA (**F**). The MGF_110-13L proteins in ASFV-infected cell lysates were detected by Western blot with mAbs 8C3 and 10E4, respectively (**G**). **, glycosylated monomer. *, unglycosylated monomer.

To express MGF_110-13L (1–131aa) in mammalian cells, BHK-21 cells were transfected with the plasmid pCAG-MGF110-13L. After limited dilution cloning and G418 selection, a stable expression cell line (BHK-110-13L) was established (Figure 2A). The recombinant MGF_110-13L protein was purified by nickel affinity chromatography from the supernatants of BHK-110-13L cells (Figure 2B). The purified recombinant protein MGF_110-13L exhibited two main bands near or above 15 kDa in SDS-PAGE analysis (Figure 2B). To survey whether cell line-expressed proteins exhibit two bands due to the presence of partially glycosylated modification, the purified protein was treated with the PNGase F enzyme and subjected to SDS-PAGE analysis. The results showed that the purified MGF_110-13L protein appeared as a single band of nearly 15 kDa after PNGase F treatment (Figure 2C). When analyzed by SDS-PAGE under the non-reducing condition (in the absence of β-mercaptoethanol), the purified MGF_110-13L protein exhibited three bands near or above 35 kDa (Figure 2D). From these results, it can be deduced that the MGF_110-13L protein secreted in the cell line is expressed as a partially glycosylated homodimer. To survey the antigenicity of the MGF_110-13L protein, three pigs were immunized with the purified protein. The sera were tested by ELISA with purified MGF_110-13L protein as the coating antigen. The results showed that MGF_110-13L-specific antibodies were developed in all immunized pigs but not in pigs in the control group (Figure 2E).

To generate MGF_110-13L-specific monoclonal antibody, purified recombinant protein was used to immunize BALB/c mice. After three immunizations, a booster was given intraperitoneally with purified soluble protein without adjuvant. After cell fusion, two hybridomas (8C3 and 10E4) were generated. The light chains of both mAbs were kappa. The isotypes of two mAbs were all tested as IgG1. mAb titers were tested by ELISA. The titers of mAbs 8C3 and 10E4 at a concentration of 1 mg/mL were above 51,200 and 102,400, respectively (Figure 2F). The Western blot results showed that the two mAbs specifically recognized the MGF_110-13L protein in lysates of ASFV-infected cells (Figure 2G). These data suggest that the two mAbs both recognized the linear epitope of the MGF_110-13L protein.

### 3.3. Epitope Mapping of mAbs

**Figure 3:**
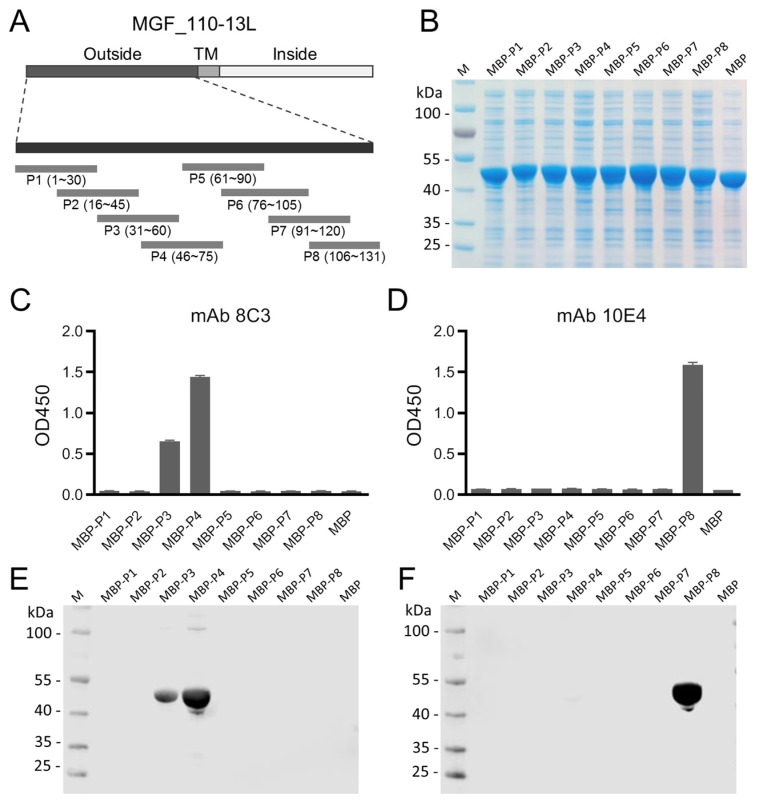
Expression of peptide fusion proteins and mapping the epitopes of mAbs. (**A**) Diagrammatic representation of the truncated overlapping short peptides that span the outer-membrane domain of the MGF_110-13L protein. (**B**) SDS-PAGE analysis of recombinant peptide fusion proteins. ELISA analysis results show that peptides P3 and P4 were recognized by mAb 8C3 (**C**), and peptide P8 was recognized by mAb 10E4 (**D**). Western blot results showed that mAb 8C3 recognized peptides P3 and P4 (**E**) and mAb 10E4 recognized peptide P8 (**F**).

To map the linear epitopes of mAbs, we designed eight partially overlapping short peptides (P1 to P8). P1 to P7 were 30 amino acids long and P8 was 26 amino acids in length. These peptides covered the entire length of the MGF_110-13L outer-membrane domain (Figure 3A). After inducing with IPTG, all eight fusion proteins were expressed in soluble form (Figure 3B). To map the epitopes of the mAbs, the fusion proteins were scanned using mAbs by ELISA. The results indicated that mAb 8C3 recognized peptides P3 and P4 (Figure 3C). However, only peptide P8 was recognized by mAb 10E4 (Figure 3D). We further conducted Western blot assays to verify the ELISA results. As expected, the Western blot results also showed that peptides P3 and P4 were recognized by mAb 8C3 (Figure 3E), and peptide P8 was recognized by mAb 10E4 (Figure 3F). These results demonstrated that two different antigenic epitopes of the MGF_110-13L protein were preliminarily identified.

### 3.4. Fine-Mapping of the Epitope of 8C3

**Figure 4:**
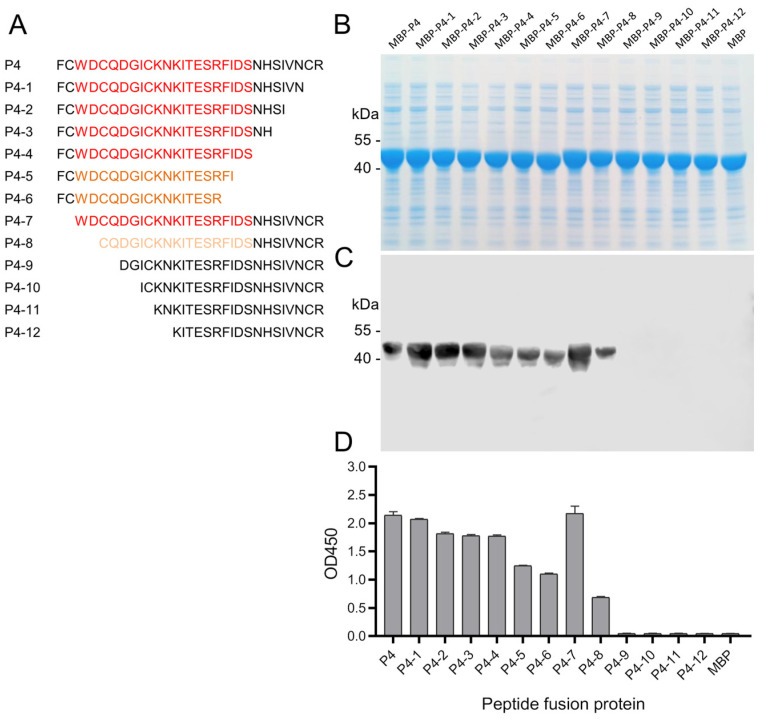
Fine-mapping of the epitope of mAb 8C3. Peptide P4 was truncated sequentially at the carboxyl or amino terminus, as shown in the schematic diagram (**A**). The MBP-peptide fusion proteins were expressed and analyzed by SDS-PAGE (**B**). The MBP-peptide fusion proteins were probed with mAb 8C3 using Western blot (**C**) and ELISA (**D**). The core sequence of the epitope of mAb 8C3 was deduced to be ^48^WDCQDGICKNKITESRFIDS^67^. The core sequences of the epitope are highlighted in red, and the truncated epitope sequences with reduced binding capacity to the mAb are shown in orange or yellow (**A**).

Though both peptides P3 and P4 were recognized by mAb 8C3, P4 showed a relatively stronger reactive signal than P3 did. So, it was supposed that the peptide ^46^FCWDCQDGICKNKITESRFIDSNHSIVNCR^75^ should contain the epitope of mAb 8C3. To determine the core sequence of the epitope of mAb 8C3, the amino acid residues of the P4 peptide ^46^FCWDCQDGICKNKITESRFIDSNHSIVNCR^75^ were deleted from the C and N terminals sequentially (Figure 4A). The peptide deletion mutants were also expressed as MBP fusion proteins (Figure 4B). The deletion mutants were scanned with mAb 8C3 by ELISA and Western blot, respectively. The results showed that the OD_450_ values did not significantly decrease when the C terminus’ eight amino acid residues were deleted. Nevertheless, the binding signals of mutants with mAb 8C3 were sharply reduced when ten or more residues were deleted from the C terminus. The reaction signal did not decrease when ^46^FC^47^ was removed from the N terminus. However, the reaction signal drastically decreased when two additional amino acid residues were eliminated. Additionally, the peptide binding reaction with mAb 8C3 almost completely disappeared when four more amino acid residues were deleted (Figure 4C,D). These results indicated that the core sequence of the epitope of mAb 8C3 was ^48^WDCQDGICKNKITESRFIDS^67^.

### 3.5. Fine-Mapping the Epitope of 10E4

**Figure 5:**
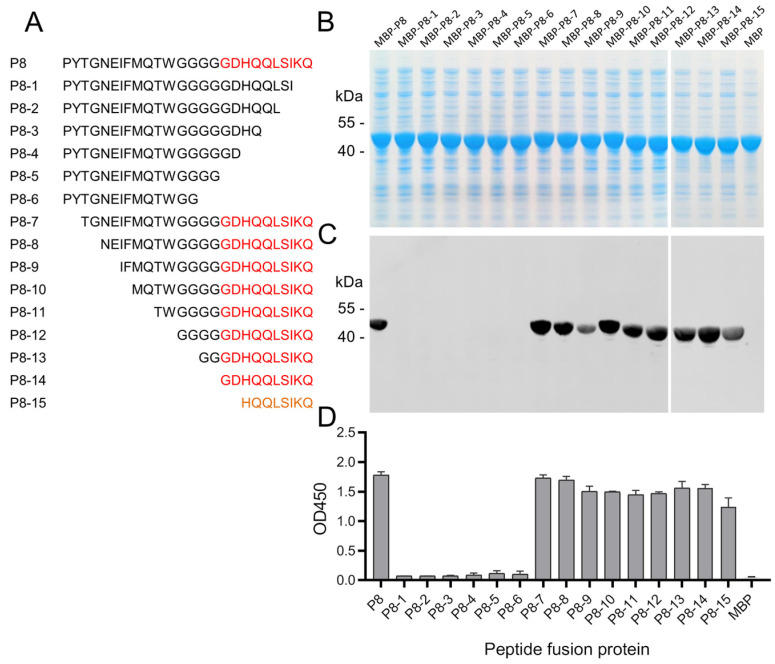
Fine-mapping of the epitope of mAb 10E4. Peptide P8 was truncated sequentially at the carboxyl or amino terminus, as shown in the schematic diagram (**A**). The MBP-peptide fusion proteins were expressed and analyzed by SDS-PAGE (**B**). The MBP-peptide fusion proteins were probed with mAb 10E4 using Western blot (**C**) and ELISA (**D**). The core sequence of the epitope of mAb 10E4 was deduced to be ^122^GDHQQLSIKQ^131^. The core sequences of the epitope are highlighted in red, and the truncated epitope sequences with reduced binding capacity to the mAb are shown in orange (**A**).

Peptide P8 was truncated sequentially from the C and N termini, respectively, in order to identify the basic unit of the epitope of mAb 10E4 (Figure 5A). The same method was used to express the peptide deletion mutants as MBP fusion proteins; all MBP-peptide fusion proteins were expressed as soluble proteins in cell lysates (Figure 5B). ELISA and Western blot were used to test the binding activities of fusion proteins with mAb 10E4. The results indicated that 16 amino acid residue deletions from the N terminus of P8 did not significantly affect the binding of peptides with mAb 10E4. However, with the deletion of only two amino acid residues (^130^KQ^131^) from the C terminus of P8, the peptide almost completely lost its ability to bind to the mAb 10E4 (Figure 5C,D). These findings showed that the linear epitope of mAb 10E4 has a core sequence of ^122^GDHQQLSIKQ^131^.

### 3.6. Immunoreactivity of the Epitope Peptides with Pig Sera

**Figure 6:**
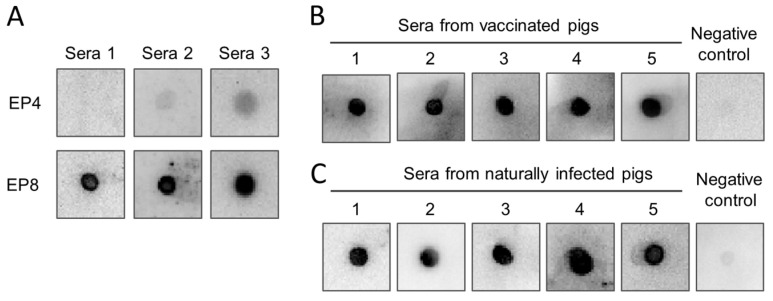
Detection of African swine fever virus antibodies with synthesized peptides. The synthesized peptides EP4 of the mAb 8C3 epitope and EP8 of the mAb 10E4 epitope were subjected to detection of epitope specific antibodies in ASFV-infected pig sera using the dot blot assay (**A**). The synthesized peptide EP8 of the mAb 10E4 epitope was further evaluated in detecting ASFV antibodies with attenuated ASFV-infected pig sera (**B**) and naturally ASFV-infected pig sera (**C**) by dot blot assay.

To survey whether the epitopes could bind with pig anti-ASFV sera, the peptides of mAb 8C3 epitope (EP4, ^48^WDCQDGICKNKITESRFIDS^67^) and 10E4 epitope (EP8, ^122^GDHQQLSIKQ^131^) were synthesized chemically and used to test the reactivity with pig sera from attenuated-ASFV-vaccinated and virulent-ASFV-strain-challenged pigs by dot blot assay. The results demonstrated that the EP8 peptide strongly reacted with all three serum samples, but the peptide EP4 could only react with two out of three serum samples weakly (Figure 6A). These results demonstrated that epitope 10E4 was an antigenic epitope of the MGF_110-13L protein. To survey the potential usage of the epitope 10E4 as an antigen for serological testing of ASFV antibodies, the synthesized peptide EP8 was further evaluated with five more attenuated ASFV-vaccinated pig sera and five naturally ASFV-infected pig sera. As the data showed, EP8 could be recognized by both artificially immunized pig sera (Figure 6B) and naturally ASFV-infected pig sera (Figure 6C).

## 4. Discussion

Although a gene-deleted modified live vaccine (MLV) has been approved in Vietnam [25,26], ASF still causes significant economic losses in the pig industry worldwide. Currently, for most of the world, well-acknowledged, safe, and effective ASF vaccines are not available. ASFV is a large and complex DNA virus with a double-stranded DNA genome that contains more than 150 ORFs. Over half of ASFV genes lack any known or predictable function. Recently, some virulence-related genes, especially host innate immune suppressor genes, were identified [27,28,29,30,31,32,33]. These findings may provide important bases for the development of gene-deletion-attenuated vaccines. Compared with attenuated live vaccines, it is generally considered that subunit vaccines have better safety. For the development of effective subunit vaccines and the development of novel serological assays, fully exploring the antigenic proteins of ASFV is necessary. Several key immunodeterminants, such as P72, p30, p54, K205R, and CD2v, have been identified. These proteins constitute potential serological assay targets and subunit vaccine components. To explore more potential antigenic proteins, in this study, we focused on predicted transmembrane proteins. Seven transmembrane proteins (C257l, I329L, I177L, EP153R, B475L, K196R, and MGF-110-13L) were transiently expressed in mammalian cells and screened with anti-ASFV pig serum. C257L, EP153R, and B475R showed weak reaction signals. EP153R was reported as a glycosylated C-type lectin-like protein, which may play crucial roles in the virulence of ASFV [34]. Deletion of EP153R attenuated the virus [35]. However, EP153R did not show good reactive activity with ASFV-positive pig serum. B475L also showed a weak reaction signal with ASFV-infected pig serum. Xu et al. recently also reported that prokaryotic-expressed B475L protein showed positive reactions with clinical ASFV-infected pig sera [36]. Here, among the seven selected transmembrane proteins, we found that MGF_110-13L showed the strongest reaction signal. These results demonstrated that MGF_110-13L is an antigenic protein of ASFV. However, the function of MGF_110-13L in inducing immune protection needs further investigation.

To express and characterize the MGF_110-13L protein, a cell line stably expressing the outer-membrane domain of the MGF_110-13L protein was generated. The purified recombinant protein exhibited two main bands by SDS-PAGE analysis. The higher molecular weight band was sensitive to PNGase F. After treatment with PNGase F, the purified protein exhibited only one main band at the same molecular weight as the lower band of the untreated protein. Furthermore, there is a predicted N-glycosylation site (^68^NHS^70^) in the outer-membrane domain of MGF_110-13L. The recombinant MGF_110-13L protein is secreted in incomplete glycosylated homodimer form. Under the non-reducing condition of SDS-PAGE, the purified protein exhibited three bands. The three bands were presumed to be glycosylated-glycosylated, glycosylated-unglycosylated, and unglycosylated-unglycosylated dimers, respectively. Whether the MGF_110-13L protein is a structural protein located in the inner membrane or outer membrane requires further investigation.

Antigenic epitopes are important characteristics of antigenic proteins. Identification of antigenic epitopes facilitates the understanding of the structure and function of antigenic proteins. To date, the antigenic epitopes of P72 [37,38], p54 [39,40], p30 [41], CD2v [42,43], and K205R [16] proteins have been reported. In the present study, using recombinant protein as an immunogen, we generated two MGF_110-13L protein-specific mAbs. To identify the epitopes of the two mAbs, a set of truncated overlapping peptides was designed and expressed as MBP fusion proteins. After being screened with mAbs by ELISA and Western blot, two epitopes located at 46 to 75 amino acids and 106 to 131 amino acids of the MGF_110-13L protein were preliminary identified, respectively. With the same strategy of expressing truncated peptides as fusion proteins, the two epitopes’ core sequences were identified as ^48^WDCQDGICKNKITESRFIDS^67^ and ^122^GDHQQLSIKQ^131^, respectively. To see if the epitopes were also recognized by sera from ASFV-infected pigs, the two epitope peptides were synthesized and tested with sera from ASFV-vaccinated and -challenged pigs. Among the two epitope peptides, peptide EP8 produced strong reaction signals, indicating that it is the antigenic epitope of MGF_110-13L. The potential application of peptide EP8 in serological testing was further investigated using five additional experimentally infected pig sera and five naturally infected pig sera. All ASFV-infected pig sera reacted positively with the peptide EP8. The findings indicate that the mAb 10E4 epitope is a potential candidate marker antigen for the serological diagnosis of ASF.

Due to the occurrence of indels or frame shift mutations, the genetic diversity of the MGF genes varied widely [44]. For MGF_110-13L, there are three variants: full-length (272–275 aa), mutants with deletion of the N-terminal outer membrane domain, and mutants with deletion of the N-terminal outer membrane domain and transmembrane domains. Up to May 11, 2024, there were 191 MGF_110-13L protein sequences in the NCBI database. Among the 191 MGF_110-13L protein sequences, there were 59 full-length MGF_110-13L protein sequences. The sequence alignment results showed that both epitope 8C3 (^48^WDCQDGICKNKITESRFIDS^67^) and epitope 10E4 (^122^GDHQQLSIKQ^131^) are highly conserved (59/59). These results suggest that the mAbs generated here could provide useful tools for further study on the MGF_110-13L protein. In addition, the epitope 10E4 has the potential to be used as an antigen in the development of serological assays. To our knowledge, this is the first study to characterize the MGF_110-13L protein epitopes. The findings of this study provide insights into future structural and functional research on the transmembrane protein MGF_110-13L.

## 5. Conclusions

In conclusion, we identified MGF_110-13L as an antigenic protein and fine-mapped the two linear epitopes of the MGF_110-13L protein of ASFV using two MGF_110-13L-specific mAbs. We also evaluated the synthesized peptide EP8 as a potential antigen for detecting ASFV antibodies by dot blot assay.

## Data Availability

The authors declare that the data supporting the findings of this study are available within the article or upon request to the corresponding authors.
